# Synergistic activation of NF-κB by TNFAIP3 (A20) reduction and UBE2L3 (UBCH7) augment that synergistically elevate lupus risk

**DOI:** 10.1186/s13075-020-02181-4

**Published:** 2020-04-25

**Authors:** Taehyeung Kim, Sang-Cheol Bae, Changwon Kang

**Affiliations:** 1 Department of Biological Sciences, Korea Advanced Institute of Science and Technology, 291 Daehak-ro, Yuseong-gu, Daejeon, 34141 Republic of Korea; 2 Department of Rheumatology, Hanyang University Hospital for Rheumatic Diseases, 222-1 Wangsimni-ro, Seongdong-gu, Seoul, 04763 Republic of Korea

**Keywords:** Autoimmune disease, Cytokine, Epistasis, Gene-gene interaction, Gene polymorphism, Inflammatory disease, Rheumatic disease, Synergistic interaction, Systemic lupus erythematosus, TNFR pathway

## Abstract

**Background:**

Systemic lupus erythematosus (SLE) is an autoimmune inflammatory rheumatic disease. SLE susceptibility is affected by multiple genetic elements, environmental factors, and their interactions. We aimed in this study to statistically and functionally characterize a gene-gene interaction (epistasis) recently documented to affect SLE risk.

**Methods:**

Two single-nucleotide polymorphisms, rs2230926 in *TNFAIP3* (*A20*) gene and rs131654 in *UBE2L3* (*UBCH7*) gene, were genotyped in all 3525 Korean participants, and their SLE risk association and epistasis were statistically analyzed by calculating odds ratio (OR), 95% confidence interval (CI), and *P* values in genotype comparisons between 1318 SLE patients and 2207 healthy controls. Furthermore, their effects on gene functions were assessed by comparatively examining separate and combined effects of TNFAIP3 and UBE2L3 knockdowns on NF-κB transcription factor activity in human cells.

**Results:**

SLE susceptibility is associated with *TNFAIP3* rs2230926 (OR = 1.9, 95% CI 1.6–2.4, *P* = 8.6 × 10^−11^) and *UBE2L3* rs131654 (OR = 1.2, 95% CI 1.1–1.4, *P* = 1.1 × 10^−4^) in a Korean population of this study. Their risk-associated alleles synergistically elevate SLE susceptibility in both multivariate logistic regression analysis (OR_interaction_ = 1.6, *P* = 0.0028) and genotype-stratified analysis (OR_interaction_ = 2.4), confirming the synergistic *TNFAIP3-UBE2L3* interaction in SLE risk. Additionally, the SLE-susceptible alleles confer decreased *TNFAIP3* expression (*P* = 1.1 × 10^−6^, *n* = 610) and increased *UBE2L3* expression (*P* = 9.5 × 10^−11^, *n* = 475), respectively, in B cell analysis of the International HapMap Project individuals with adjustment for ethnicity. Furthermore, when compared with TNFAIP3 non-knockdown and UBE2L3 knockdown in human HeLa cells, TNFAIP3 knockdown and UBE2L3 non-knockdown synergistically increase three cytokines, CCL2, CXCL8 (IL8), and IL6, all regulated by NF-κB in the human TNFR signaling pathway.

**Conclusions:**

A synergistic interaction between *TNFAIP3* and *UBE2L3* genes is observed in SLE risk, as being evident in comparison of genotype distributions between SLE patients and controls. Additionally, the synergistic gene-gene interaction is functionally validated, as TNFAIP3 reduction and UBE2L3 augment exert synergism in activation of NF-κB and subsequent induction of inflammatory cytokines. Accordingly, SLE inflammation and risk could be synergistically alleviated by TNFAIP3 upregulation and UBE2L3 downregulation.

## Background

Systemic lupus erythematosus (SLE) [MIM 152700], the most common type of lupus, is an autoimmune inflammatory rheumatic disease. SLE is a complex disease, as its susceptibility is affected by multiple genetic and nongenetic factors. Its genetic heritability is 44–66% [1, 2], but all the SLE-associated genetic elements discovered so far can explain roughly only 24% [3]. The missing genetic heritability can be partly attributed to non-linear effects arising from gene-gene interactions, among others [4].

The genetic interaction where the phenotypic effect of a gene allele depends on the presence of one or more modifier gene alleles is referred to as gene-gene interaction, or epistasis [5]. This phenotypic dependence could result from an underlying functional interaction of the genes. Some epistases have been identified for SLE, but no functional validation has been achieved to explain how the interacting genetic variants can influence SLE development.

Recently, an interaction between two NF-κB modulator genes in SLE risk has been found in Han Chinese [6] and European populations [7]. The interaction was synergistic between *TNFAIP3* gene encoding TNF alpha-induced protein 3 (A20) and *UBE2L3* gene encoding ubiquitin-conjugating enzyme E2 L3 (UBCH7). However, the examined single-nucleotide polymorphisms (SNPs) were different in the two case-control studies, reporting very different *P* values for the SLE risk interaction, *P* = 3 × 10^−14^ versus 0.04 [6, 7]. In this study, we reexamined their gene-gene interaction in Korean SLE patients and controls, and explored their functional consequences in human cells.

SNPs of the two genes are associated with multiple autoimmune diseases including SLE [8], and their proteins are involved in diverse regulations of NF-κB transcription factor activity [9–17]. TNFAIP3 plays a potent suppressor of NF-κB pathways for immune homeostasis using its diverse ubiquitin-related activities. First, TNFAIP3 is an E3 ubiquitin ligase that catalytically modifies substrate proteins with a Lys-48-linked ubiquitin chain using a C-terminal domain (K48ub writer) [9, 10]. Second, TNFAIP3 is a deubiquitinating enzyme that catalytically removes a Lys-63-linked ubiquitin chain from modified proteins using the N-terminal ovarian tumor domain (K63ub eraser) [9]. Third, TNFAIP3 is a ubiquitin receptor that non-catalytically binds a Met-1-linked ubiquitin chain of partner proteins using a zinc finger domain (M1ub reader) [11–13].

**Fig. 1:**
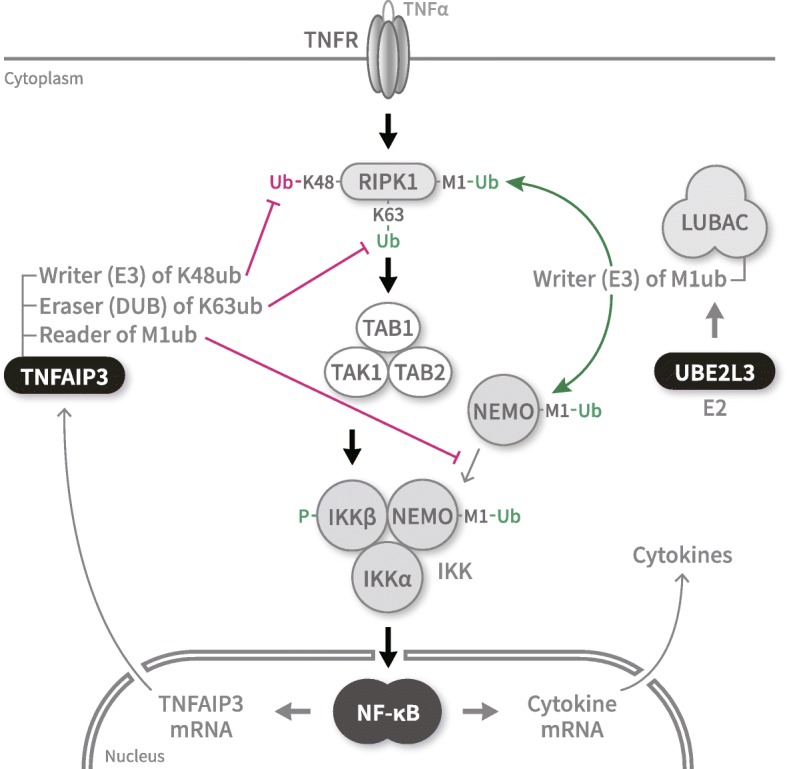
Participation of TNFAIP3 and UBE2L3 in regulation of NF-κB activity under TNFα signaling. The human TNFR pathway transduces a signal of extracellular TNFα through cytoplasmic IKK to latent transcription factor NF-κB for cytokine induction in the nucleus, among others. TNFAIP3 negatively regulates NF-κB (marked by red arrows) (1) by mediating RIPK1 degradation via K48 ubiquitylation (E3), (2) by inactivating RIPK1 via K63 deubiquitylation (DUB), and (3) by inhibiting IKK via binding ubiquitylated M1 of NEMO. In contrast, UBE2L3 (E2) positively regulates NF-κB (marked by green arrows) by helping LUBAC (E3) to activate RIPK1 and MEMO via M1 ubiquitylation

In the human TNFR signaling pathway (Fig. 1), TNFAIP3 inhibits NF-κB activation in multiple ways. First, TNFAIP3 mediates degradation of RIPK1. As a K48ub writer, TNFAIP3 attaches K48ub to RIPK1 so that RIPK1 is degraded by proteasome [9]. Second, TNFAIP3 inactivates RIPK1, which regulates IκB kinase (IKK) [14]. As a K63ub eraser, TNFAIP3 detaches K63ub from RIPK1 so that RIPK1 cannot interact with other modulators [9]. Third, TNFAIP3 inhibits IKK activation. As an M1ub reader, TNFAIP3 binds M1ub of NEMO (IKKγ), which forms IKK complex with IKKα and IKKβ, and inhibits the phosphorylation of IKKβ by TAK1 [11–13]. All these activities of TNFAIP3 disable IKK activation in the cytoplasm to reduce NF-κB activity in the nucleus.

On the other hand, UBE2L3 is an E2 ubiquitin-conjugating enzyme that pairs up with an E3 ubiquitin ligase of HECT (homologous to the E6AP carboxyl terminus) type or RBR (ring between ring) family, but not of ring type, for catalytic ubiquitylation [15]. In the human TNFR pathway (Fig. 1), UBE2L3 E2 enzyme provides activated ubiquitin to LUBAC E3 enzyme (RBR family) for M1 ubiquitylation of RIPK1 and NEMO [16, 17]. All these activities of UBE2L3 enable the cytoplasmic IKK activation to enhance the nuclear NF-κB activity.

Thus, TNFAIP3 and UBE2L3 participate in the same pathway but exert opposite effects on sequential activation of IKK and NF-κB and subsequent induction of inflammatory cytokines (Fig. 1). In principle, two genes functionally participating in one pathway can interact with each other in affecting disease susceptibility even without their direct protein-protein interaction [18]. Accordingly, we aimed in this study to validate whether TNFAIP3 reduction and UBE2L3 augment influence each other’s effect on NF-κB transcription factor activation for cytokine induction.

## Materials and methods

### Human subjects and SNP genotyping

Korean participants were recruited at six university-affiliated hospitals in Seoul, Daejeon, and Daegu, Republic of Korea, in a study approved by the Institutional Review Board of Hanyang University Medical Center, and all provided written informed consent [19, 20]. Upon approval from the Institutional Review Board of Korea Advanced Institute of Science and Technology, peripheral blood samples from all subjects were genotyped for SNPs using the MassARRAY® system from Sequenom [20, 21].

### Statistical analyses

Logistic regression analysis of the PLINK v1.07 program [22] was performed to calculate odds ratio (OR), 95% confidence interval (CI), and *P* values for SLE risk association and epistasis with adjustment for age and gender of participants by using the -logistic and -covar options [20, 23–25]. SNP association was corrected for multiple testing.

For testing multiplicativity in gene-gene interaction, we added an interaction term in logistic regression: logit{P(disease = 1)|SNP_1_ = A, SNP_2_ = B, interaction SNP_1_ × SNP_2_ = AB, age = C, and gender = D} = *β*_0_ + *β*_1_A + *β*_2_B + *β*_3_AB + *β*_4_C + *β*_5_D. When OR of the risk-associated genotype in one SNP is OR_1_ = e^*β*^_1_, that in the other SNP is OR_2_ = e^*β*^_2_, and that in both SNPs is OR_1&2_ = e^*β*^_3_, a multiplicative interaction between the two genes is represented by OR_int_ = OR_1&2_/(OR_1_ × OR_2_) [18, 26].

We used CRAN R package “epiR” to calculate an attributable proportion (AP) due to interaction, 95% confidence interval (CI), and *P* value in Rothman’s additive model [27]. The combined relative risk of the two SNPs (RR_11_) was estimated from their individual relative risks (RR_10_ and RR_01_) by RR_11_ = RR_10_ + RR_01_ – 1, as the presence of risk allele is denoted by subscript 1 and its absence by subscript 0. AP is a derivative measure of the relative excess risk caused by additive interaction: AP = (RR_11_ – RR_10_ + 1)/RR_11_ [26, 28].

### Gene knockdowns

Human HeLa cells purchased from the American Type Culture Collection were maintained in Dulbecco’s modified Eagle’s medium supplemented with 10% fetal bovine serum by incubation at 37 °C in 5% CO_2_ atmosphere. The cells were seeded in 6-well plates at 1 × 10^5^ cells/well, and 24 h later transfected with one or two small interfering RNAs (siRNAs) or a control of 15 nM each. At 72 h after transfection, the cells were treated with 10 ng/ml of human recombinant TNFα, 210-TA from R&D System, for 0, 2, 4, 8, and 12 h.

Three siRNAs, HS S110861 and HS S110862 from Thermo Fisher Scientific and SI00086989 from Qiagen, were individually used for TNFAIP3 knockdown and are denoted here by siA20-1, siA20-2, and siA20-3, respectively. An anti-UBE2L3 siRNA SI05191242 from Qiagen, denoted here by siUBE, was used to knockdown UBE2L3 [17]. AccuTarget Control siRNA SN-1002 from Bioneer, denoted here by siCon, served as a negative control.

### Protein and RNA quantifications

The whole-cell lysates were obtained using the passive lysis buffer from Promega. The protein extracts (50 μg) were subjected to western blotting using anti-TNFAIP3 antibody 59A426 from Merck Millipore, anti-UBE2L3 antibody 3848S from Cell Signaling Technology, and anti-β-actin antibody SC-1616-R from Santa Cruz Biotechnology.

Total RNA was isolated from cells using the RNAspin kit from iNtRON Biotechnology and used for cDNA library construction using oligo-dT primer and ImProm-II reverse transcriptase from Promega. Quantitative polymerase chain reaction (qPCR) was performed with SYBR green fluorescent dye using CFX96™ real-time qPCR detection system from Bio-Rad Lab and gene-specific primers such as GTCTCCTCTGACTTCAACAGCG (forward) and ACCACCCTGTTGCTGTAGCCAA (reverse) for *GAPDH* [29], GAAAGTCTCTGCCGCCCTT and ATTGATTGCATCTGGCTGAGCG for *CCL2* [7], AGCTCTGTCTGGACCCCAAG and GAATTCTCAGCCCTCTTCAAAAAC for *CXCL8* [30], and CCCCCAGGAGAAGATTCCAA and GCTGCTTTCACACATGTTACTCTTG for *IL6* [31]*.* Relative mRNA levels were estimated using the comparative Ct method, ΔΔCt method [32].

## Results

### *TNFAIP3-UBE2L3* interaction in SLE risk

In this study, a total of 3525 Korean participants were all genotyped for *TNFAIP3* rs22230926 and *UBE2L3* rs131654 SNPs, which had shown the lowest *P* value among the SNPs of each respective gene locus in three previous genome-wide association studies on Asian SLE susceptibility [19, 33, 34]. For example, their *P* values were 1 × 10^−17^ and 3 × 10^−16^, respectively, in a Han Chinese population [33]. The SNP genotype distributions among the control subjects were in Hardy-Weinberg equilibria.

The SNP genotypes were statistically compared between 1318 SLE patients (34.4 ± 12.4 years; 93% female) and 2207 healthy controls (40.7 ± 15.4 years old; 77% female) using logistic regression analysis (Table 1). Parts of these Korean samples previously demonstrated no genetic stratification in principal component analyses [19, 35], but the case and control groups of this study were different in age distribution and gender ratio, so the logistic regression was adjusted for age and gender of the participants. SLE susceptibility is associated with both *TNFAIP3* rs2230926 (OR = 1.9, 95% CI 1.6–2.4, *P* = 8.8 × 10^−11^) and *UBE2L3* rs131654 (OR = 1.2, 95% CI 1.1–1.4, *P* = 1.1 × 10^−4^) SNPs, as their *P* values are much lower than a significance threshold of Bonferroni correction for 2-SNP testing, *α* = 0.05/2 = 0.025.

**Table 1:** Genotype distributions and SLE risk associations of *TNFAIP3* and *UBE2L3* SNPs

	Controls	Cases	SLE risk association^b^	
SNP major>minor^a^	*nn*/*nr*/*rr*^a^	*nn*/*nr*/*rr*^a^	OR (95% CI)	*P*
*TNFAIP3* rs2230926 *T*>***G***	1989/213/5	1069/240/9	1.9 (1.6–2.4)	8.8 × 10^−11^
*UBE2L3* rs131654 ***T***>*G*	572/1116/519	274/669/375	1.2 (1.1–1.4)	1.1 × 10^−4^

*SLE* systemic lupus erythematosus, *SNP* single-nucleotide polymorphism, *OR* odds ratio, *CI* confidence interval

^a^Major alleles are more frequent than minor alleles in controls. Shown in bold are the risk-associated alleles (*r*), which are more frequent in SLE cases than healthy controls. The nonrisk-associated alleles (*n*) are more frequent in controls than cases

^b^Using logistic regression with adjustment for age and gender of the participants

Additionally, the gene-gene interaction was examined using this SNP pair. A multivariate logistic regression analysis with their interaction term according to a multiplicative model [26] showed a synergistic interaction with the two SNPs (OR_int_ = 1.6, *P* = 0.0028), indicating a synergistic interaction between *TNFAIP3* and *UBE2L3* genes in conferring SLE risk. Furthermore, this SNP pair revealed a similar interaction (AP of interaction = 0.42; 95% CI 0.18–0.66, *P* = 0.049) additionally in Rothman’s additive model based on additivity of the individual relative risks [27].

This *TNFAIP3*-*UBE2L3* interaction was further tested by using genotype-stratified analysis. According to the genotypes of the two SNPs, all study subjects are stratified into six rather than nine subgroups in Table 2, as heterozygotes in the *TNFAIP3* SNP are grouped together with homozygotes of the risk-associated minor allele *G*, which are too few to form a subgroup alone. Thus, the *TNFAIP3* SNP genotypes are classified into risk-associated allele carriage (*R*) and non-carriage (*N*), while the *UBE2L3* SNP genotypes are denoted by *nn*, *nr*, and *rr*, comprising of the risk-associated major allele *T* (*r*) and the nonrisk-associated minor allele *G* (*n*).

**Table 2:** Genotype-stratified analysis of the *TNFAIP3-UBE2L3* interaction in SLE risk

Subgroup	*N*/*nn*	*N*/*nr*	*N*/*rr*	*R*/*nn*	*R*/*nr*	*R*/*rr*
Genotype^a^	*TT*/*GG*	*TT*/***T****G*	*TT*/***TT***	(*T****G***+***GG*)**/*GG*	(*T****G***+***GG*)**/***T****G*	(*T****G***+***GG*)**/***TT***
Controls	509	999	481	63	117	38
Cases	232	540	297	42	129	78
*P*^b^	–	0.13	0.0090	0.21	1.6 × 10^−7^	8.4 × 10^−11^
OR^b^	1.0	1.2	1.3	1.3	2.3	4.1
OR_int_	–	–	–	–	1.5	2.4

*SLE* systemic lupus erythematosus, *OR* odds ratio, *CI* confidence interval

^a^In the genotypes of *TNFAIP3* rs2230926/UBE2L3 rs131654, shown in bold are their risk-associated alleles, which are more frequent in patient cases than healthy controls

^b^Using logistic regression with adjustment for age and gender of the participants

ORs of the *R*/*rr*, *R*/*nn*, and *N*/*rr* subgroups are 4.1-, 1.3-, and 1.3-fold greater than the *N*/*nn* subgroup, yielding OR_int_ = 4.1/(1.3 × 1.3) = 2.4, a synergistic interaction. That is, the combined effect of *R* and *rr* is 2.4-fold greater than the sum of their separate effects versus *N* and *nn*. Additionally, a comparison among the ORs for *R*/*nr*, *R*/*nn*, and *N*/*nr* subgroups versus *N*/*nn* subgroup yields a 1.5-fold increased combined effect, OR_int_ = 2.3/(1.3 × 1.2) = 1.5.

A similar gene-gene interaction was recently found in Han Chinese susceptibility to SLE with *TNFAIP3* rs2230926 and *UBE2L3* rs463426 SNPs (*P* = 3.1 × 10^−14^) in generalized multifactor dimensionality reduction analysis [6]. Another similar interaction was found in European susceptibility to SLE with *TNFAIP3* rs80126770 and *UBE2L3* rs140490 SNPs (OR_int_ = 1.3), but the *P* value was not very low (*P* = 0.039) [7]. Although the documented SNP pairs were all different, the three independent epistasis studies appear to support for the *TNFAIP3*-*UBE2L3* interaction in multiple ethnic populations.

### Expression quantitative trait loci (eQTL) of *TNFAIP3* and *UBE2L3*

**Fig. 2:**
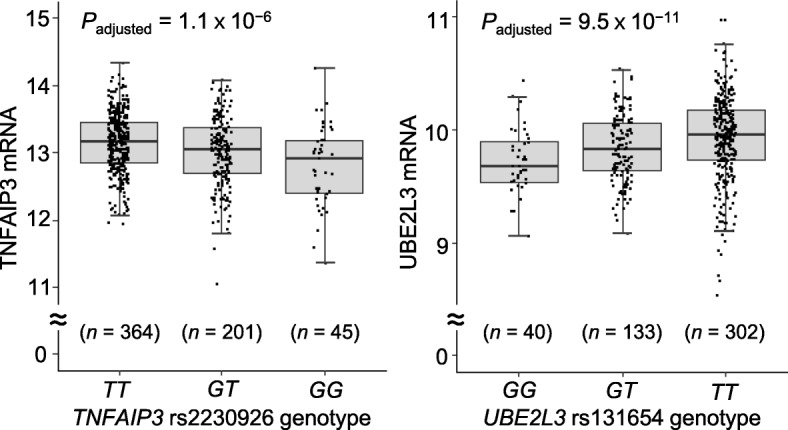
Association of *TNFAIP3* and *UBE2L3* gene expressions with two SNPs. The genotype and mRNA data of 726 EBV-transformed B cell samples of the HapMap Project were obtained from the Array Express Database (series accession number E-MTAB-264). *TNFAIP3* rs2230926 genotypes of 610 individuals (left graph) and *UBE2L3* rs131654 genotypes of 475 individuals (right graph) were obtained from the Ensemble genome browser. Correlations between genotypes and gene expressions were analyzed using linear regression with adjustment for ethnic backgrounds of the samples

It was then examined whether the SLE risk-associated alleles confer increase or decrease in their respective gene expression. First, we used gene expression and genotype data of Epstein-Barr virus (EBV)-transformed B cell lines derived from the International HapMap Project participants [36] to calculate eQTL *P* values with an additive linear function with adjustment for ethnic background using R MatrixEQTL package [37, 38]. The SLE risk-associated allele *G* of rs2230926 is associated with decreased TNFAIP3 mRNA (*P* = 1.1 × 10^−6^) in 610 individuals (Fig. 2, left). In contrast, increased UBE2L3 mRNA is associated with the risk-associated allele *T* of rs131654 (*P* = 9.5 × 10^−11^) in 475 individuals (Fig. 2, right). Thus, the SLE risk-associated variants are associated with decreased TNFAIP3 and increased UBE2L3, both elevating NF-κB transcription factor activity.

Second, we analyzed the Genotype-Tissue Expression Project data (V8 release) [39]. *TNFAIP3* mRNA is not associated with rs2230926 in any of the 47 analyzed tissues, although it is associated with some other SNPs in subcutaneous adipose and brain hippocampus tissues. *UBE2L3* mRNA is marginally associated with rs131654 in skeletal muscle (*P* = 0.0008), whole blood (*P* = 0.0023), subcutaneous adipose (*P* = 0.012), tibial artery (*P* = 0.019), and esophagus muscularis (*P* = 0.045) tissues, as their *P* values are less than 0.05 but higher than a gene-wise significance threshold for each tissue.

### Functional assay of *TNFAIP3-UBE2L3* interaction

TNFAIP3 and UBE2L3 enzymes participate together in the human TNFR [9–13, 40, 41], IL1R [41, 42], NOD2 [43, 44], RIG1 [45, 46], and TLR4 [42, 43] immune pathways, among others. Their cellular and molecular functions have been well characterized in the TNFR pathway, which was chosen in this study for examining their concurrent effects on NF-κB activity for cytokine induction. Transcript mRNAs produced from three NF-κB-dependent cytokine genes, *CCL2*, *CXCL8* (*IL8*), and *IL6*, were quantified in TNFα-treated HeLa cells.

In order to compare the effects of relatively higher and lower levels of TNFAIP3 and UBE2L3 on NF-κB activity, four different HeLa cell derivatives were constructed using combinations of TNFAIP3 and UBE2L3 knockdowns. The first cell line without TNFAIP3 knockdown but with UBE2L3 knockdown has relatively high TNFAIP3 and low UBE2L3, and serves as a reference. The second cell line with both knockdowns has low TNFAIP3 and UBE2L3, and provides the TNFAIP3 reduction effect compared with the reference. The third without any knockdown has high TNFAIP3 and UBE2L3, and shows the UBE2L3 augment effect. The fourth with TNFAIP3 knockdown alone has low TNFAIP3 and high UBE2L3, and reveals the combined effect of TNFAIP3 reduction and UBE2L3 augment.

**Fig. 3:**
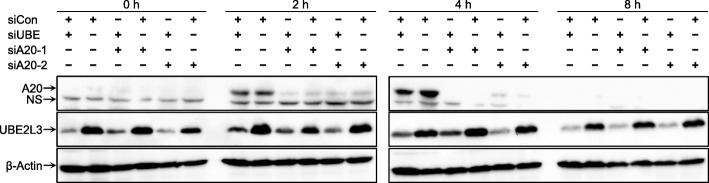
Knockdowns of TNFAIP3 and UBE2L3 for functional assays. After HeLa cells were transfected with various combinations of siRNAs (siCon, siUBE, and siA20-1 or siA20-2), protein levels of TNFAIP3 (A20), UBE2L3, and β-actin were measured using western blotting at the indicated time points. NS means nonspecific band

TNFAIP3 is effectively knocked down by siA20-1, siA20-2, or siA20-3 separately, although they target different sequences in TNFAIP3 mRNA (Fig. 3). The UBE2L3 knockdown is effective with siUBE, as its efficiency and specificity have been demonstrated under the same conditions as this study, i.e., in HeLa cells under TNFR activation [17]. Furthermore, siCon is an adequate negative control as it does not affect *CCL2*, *CXCL8*, or *IL6* expression.

### Synergistic activation of NF-κB

**Fig. 4:**
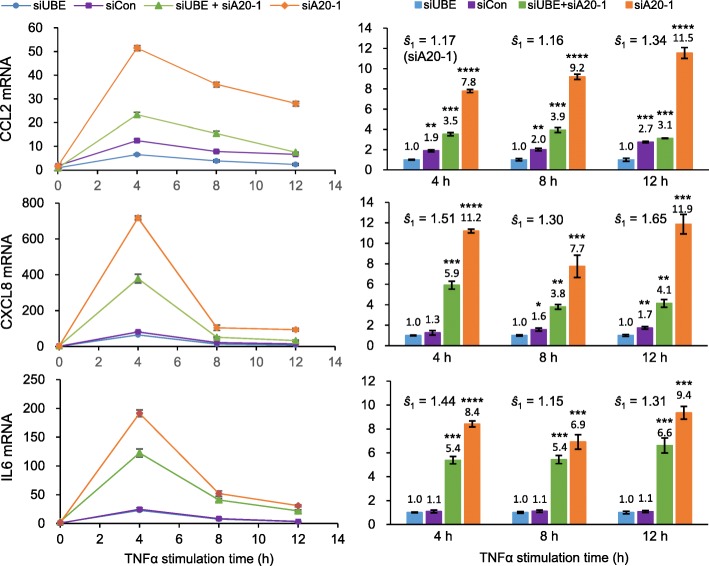
Functional assays for synergistic induction of NF-κB-mediated cytokine expressions. Four HeLa cell-line derivatives with relatively high or low TNFAIP3 and high or low UBE2L3 levels were treated with TNFα for varying periods. Cellular levels of CCL2, CXCL8 (IL8), and IL6 mRNAs were separately measured using qPCR as normalized against GAPDH mRNA. The mRNA levels were measured in triplicate samples and each sample by triplicate qPCR experiments. The nine measurements were averaged per cytokine at each time point. *P* values for comparisons of cytokine expressions between the reference cell line, transfected with siUBE and siCon, and each of the other three derivatives were calculated in unpaired *t* tests, * *P* ≤ 0.05, ** *P* ≤ 0.01, *** *P* ≤ 0.001, **** *P* ≤ 0.0001. The synergy (*ŝ*) is calculated as *ŝ* = combined effect ÷ (TNFAIP3 reduction effect × UBE2L3 augment effect)

From the four cell lines constructed using different combinations of siA20-1, siUBE, and siCon, the three cytokine mRNAs were quantified in a time-course manner at 0, 4, 8, and 12 h after TNFα treatment (Fig. 4). Either TNFAIP3 reduction or UBE2L3 augment alone increases all three cytokine mRNAs. The combined effect of TNFAIP3 reduction and UBE2L3 augment on each cytokine (fold change of mRNA) is greater than the sum of their individual effects at any of the three time points, indicating their consistent synergistic effects.

When the synergy (*ŝ*) is calculated as *ŝ* = combined effect ÷ (TNFAIP3 reduction effect × UBE2L3 augment effect), the synergy on *CCL2* or *CXCL8* expression is higher at 12 h (1.3- or 1.7-fold, respectively) than at 4 or 8 h after TNFα treatment. By contrast, the synergy on *IL6* expression is maximal at the earliest time point, 4 h (1.4-fold). Thus, the synergy peaks with *IL6* expression sooner than *CCL2* or *CXCL8* expression.

**Fig. 5:**
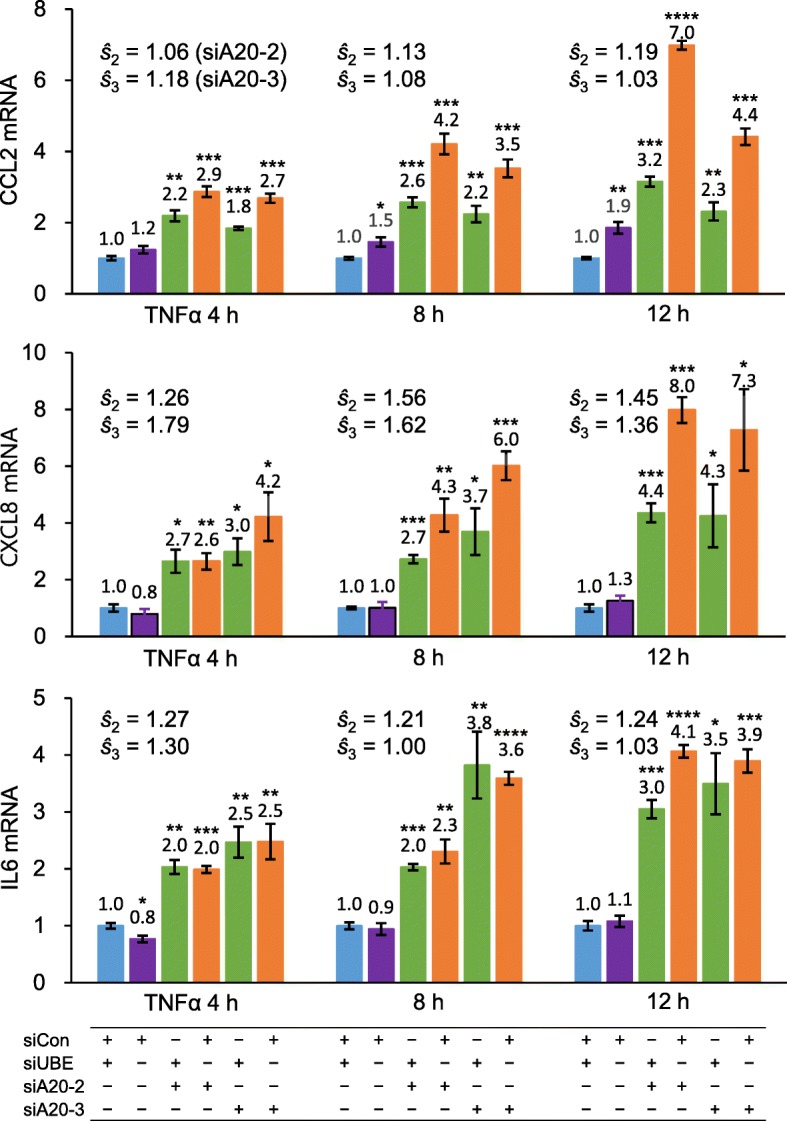
Reproduced functional assays for synergistic cytokine induction. In place of siA20-1, two other anti-TNFAIP3 siRNAs with different target sequences, siA20-2 and siA20-3, were used in HeLa cells to demonstrate the absence of off-target effects on synergistic induction of NF-κB-mediated cytokine expressions. After treatment with TNFα for varying periods, CCL2, CXCL8, and IL6 mRNAs were measured against GAPDH mRNA using qPCR. See the legend of Fig. 4

This set of experiments was repeated using two other anti-TNFAIP3 siRNAs, siA20-2 and siA20-3, separately in place of siA20-1. Although the interaction extents vary with the siRNAs, their effects on *CCL2*, *CXCL8*, and *IL6* expressions are reproduced (Fig. 5), excluding possible off-target effects of anti-TNFAIP3 siRNAs, and confirming the synergistic effects of TNFAIP3 reduction and UBE2L3 augment on their activation of NF-κB transcription factor and subsequent elevation of inflammatory cytokines.

## Discussion

Here, we report the first functional validation of gene-gene interaction in conferring susceptibility to SLE, an autoimmune inflammatory rheumatic disease. First, a synergistic interaction between *TNFAIP3* and *UBE2L3* in SLE risk is evident with their respective SNPs, rs2230926 and rs131654 (Table 2). Second, their risk-associated alleles are associated with decreased TNFAIP3 mRNA and increased UBE2L3 mRNA, respectively (Fig. 2). Third, while TNFAIP3 reduction and UBE2L3 augment separately elevate NF-κB transcription factor activity, they together additionally show a synergism in NF-κB activation for cytokine induction (Figs. 4 and 5).

The synergistic activation of NF-κB in the nucleus can be ascribed to a mechanistic connection between TNFAIP3 and UBE2L3 in the cytoplasm. In the human TNFR pathway (Fig. 1), an extracellular activation signal is transduced to latent transcription factor NF-κB through a series of sequential events in the cytoplasm: activation of IKK, inactivation of IκB, and activation and nuclear import of NF-κB [11–13]. There is another positive route for NF-κB activation: UBE2L3 E2 enzyme supplies activated ubiquitin to LUBAC E3 enzyme for linear ubiquitylation of NEMO and subsequent activation of IKK [17, 47]. Then, activated NF-κB induces *TNFAIP3* expression to exert a negative feedback on NF-κB activation, among others. Accordingly, with higher UBE2L3, NF-κB could be less inhibited by TNFAIP3.

The synergistic effect on NF-κB is evident when UBE2L3 stimulation overrides TNFAIP3 inhibition (Figs. 4 and 5). UBE2L3 augment increases the three cytokines 1.1–2.7-fold at 4, 8, and 12 h time points in comparisons of the two cell lines without TNFAIP3 knockdown. The same cytokines increase 1.3–3.7-fold in comparisons of the two cell lines with TNFAIP3 knockdown. Thus, the UBE2L3 augment effect tends to be greater with low TNFAIP3 than with high TNFAIP3, although the ranges overlap.

The functional consequences of this gene-gene interaction in NF-κB signaling need to be replicated in human immune cells instead of cervical HeLa cells used in this study. As a key modulator of diverse immune and inflammatory responses [48], NF-κB induces inflammatory cytokines and chemokines in innate immune cells, modulates inflammatory T cell functions [49, 50], and regulates inflammasome activation [51], among others. Thus, deregulated NF-κB activation is associated with multiple chronic inflammatory diseases including SLE.

For example, NF-κB promotes proliferation and survival of B cells [52], and is constitutively activated in peripheral B cells from SLE patients [53]. Elevation of *GLK* expression in SLE patients activates NF-κB signaling under T cell receptor, and is correlated with disease severity [54]. FcγRIIB-deficient mice with SLE-like symptoms have elevated NF-κB function and immunogenicity in their splenic dendritic cells [55].

While NF-κB is ubiquitously involved in diverse immune cell types, both TNFAIP3 and UBE2L3 participate in B cell differentiation, and SLE risk-associated alleles of their genes affect NF-κB activation in B cells. B cell-specific ablation of *Tnfaip3* in mice exaggerates NF-κB response to CD40-induced signals, leading to SLE-like features, including elevation of germinal center B cells and plasma cells, production of autoreactive immunoglobulins, and immune complex deposits [56–58]. UBE2L3 augment increases basal and CD40-stimulated NF-κB activation in CD19^+^ B cells. An SLE risk-associated allele of *UBE2L3* is associated with increased plasmablasts and plasma cells in SLE patients [7]. Accordingly, B cells could be one of the immune cells where the said synergism is connected with SLE phenotypes.

*TNFAIP3* rs2230926 SNP affects *TNFAIP3* gene expression (Fig. 2, left). This SNP is perfectly correlated (*r*^2^ = 1.0) with another SLE risk-associated dinucleotide polymorphism, rs148314165 and rs200820567 (*TT*>*A*) located in a *TNFAIP3-*downstream enhancer, according to the Asian population data of the 1000 Genomes Project. The risk-associated minor allele *A* renders the enhancer to lack NF-κB binding and reduce *TNFAIP3* expression in EBV-transformed B cells and HEK293T cells [59–61].

*UBE2L3* rs131654 SNP also affects *UBE2L3* gene expression (Fig. 2, right). This SNP is highly correlated with two other SLE risk-associated SNPs, rs140490 in the promoter (*r*^2^ = 0.76) and rs7444 in the 3′ untranslated region (*r*^2^ = 0.74) according to the Asian data of the 1000 Genomes Project. Their risk-associated alleles have been associated with increased UBE2L3 mRNA or protein in EBV-transformed B cells, CD19^+^ B cells, CD14^+^ monocytes [7, 62].

## Conclusions

TNFAIP3 reduction and UBE2L3 augment together confer synergistic activation of NF-κB transcription factor and subsequent synergistic induction of inflammatory cytokines. This functional demonstration supports for the *TNFAIP3*-*UBE2L3* gene-gene interaction that is statistically observed in synergistic elevation of SLE risk. Accordingly, inflammation in SLE could be synergistically reduced by concurrent upregulation of TNFAIP3 and downregulation of UBE2L3, and an inclusion of this synergistic genetic effect in weighted genetic risk score would improve SLE risk prediction.

## Data Availability

The datasets used and/or analyzed during the current study are available from the corresponding author on reasonable request.
